# Antibiotics to Prevent Surgical Site Infection (SSI) in Oral Surgery: Survey among Italian Dentists

**DOI:** 10.3390/antibiotics10080949

**Published:** 2021-08-06

**Authors:** Marco Lollobrigida, Gianluca Pingitore, Luca Lamazza, Giulia Mazzucchi, Giorgio Serafini, Alberto De Biase

**Affiliations:** Department of Oral and Maxillofacial Sciences, Sapienza University of Rome, 00161 Rome, Italy; pingitore.1631372@studenti.uniroma1.it (G.P.); luca.lamazza@uniroma1.it (L.L.); mazzucchi.1536500@studenti.uniroma1.it (G.M.); giorgio.serafini@uniroma1.it (G.S.); alberto.debiase@uniroma1.it (A.D.B.)

**Keywords:** antibiotics, antibiotic prophylaxis, surgical site infection, postoperative infection, oral surgery, antimicrobial resistance, antibiotic resistance

## Abstract

The benefit of an antibiotic prophylaxis for most oral surgical procedures is controversial. The aim of this study was to collect information on the prescribing habits of a sample of Italian dentists with respect to the role of antibiotic prophylaxis in preventing surgical site infections (SSI). An anonymous questionnaire was prepared and made accessible online by sharing a Google Forms link. General anagraphic data and educational background information were collected to obtain a profile of the participants. Different clinical scenarios were then proposed, with the participants asked to choose whether they would prescribe an antibiotic prophylaxis and with which dosage regimens. In total, 169 dentists participated in the questionnaire and the obtained data were assessed through a percentage report. The results showed a substantial agreement in antibiotics prescription, but only in a limited number of clinical scenarios, such as deciduous teeth extraction or simple extractions in healthy adult patients. Discordant responses were found for several clinical cases, particularly for cases of comorbidities, surgical or multiple extractions, implant placement and abscess drainage. The answers obtained from the survey sample were notably heterogeneous, indicating that the choice to prescribe an antibiotic prophylaxis to prevent SSIs is often discretionary. Moreover, the dosage regimen of prophylaxis is also controversial. The results of this study demonstrate the need for specific guidelines on antibiotics in dentistry and, specifically, on antibiotic prophylaxis in oral surgery. Such guidelines would help to avoid unnecessary prescriptions.

## 1. Introduction

Antibiotics are among the most common medications prescribed by dentists, both as a therapy of odontogenic infections and as prophylaxis to prevent surgical site infections (SSI) [1]. Although antibiotic therapies are dictated by objective infectious diseases, there are no specific guidelines concerning the use of antibiotics to prevent SSIs following oral surgical procedures. Therefore, the prescription of antibiotic prophylaxis (AP) is often left to the personal experience and considerations of dental clinicians. More often, antibiotics are prescribed either as standard practice for any kind of oral surgical procedure [2], or as a form of defensive medicine [3]. This leads to a general overprescription and contributes to an increased risk of antimicrobial resistance (AMR) in the population.

Perioperative antibiotic prophylaxis is defined as the administration of antibiotics before or during a surgical procedure to prevent SSI episodes [4]. Some authors distinguish between primary prophylaxis, secondary prophylaxis and eradication. Primary prophylaxis is defined as the prevention of an initial infection; secondary prophylaxis is defined as the prevention of the recurrence or reactivation of a pre-existing infection; and eradication refers to the elimination of a colonized organism to prevent the development of an infection [5]. According to these case definitions, most APs in dentistry consist of primary or secondary prophylaxis.

Although the European Centre for Disease Prevention and Control (ECDC) did not address oral and maxillofacial surgery in its report [4], the guidelines developed by the American Society of Health-System Pharmacists (ASHP), the Infectious Diseases Society of America (IDSA), the Surgical Infection Society (SIS), and the Society for Healthcare Epidemiology of America (SHEA) dedicate one section to head and neck surgery, though this is not specific to the dental field [5]. According to these guidelines, a head and neck procedure involving the incision of the oral or pharyngeal mucosa is to be considered as clean-contaminated; thus, an AP is recommended for most of these procedures. However, oral surgical procedures are less invasive and, even in clean-contaminated conditions, do not usually require antibiotic prophylaxis.

Different factors can be related to an increased risk of SSI, including procedure- and patient-related factors. The former comprises the duration and invasiveness of the procedure, bone instrumentation, the use of biomaterials, the surgical technique and sterilization practices. Patient-related factors include age, nutritional status, diabetes, smoking, obesity, coexisting infections or contaminations, and altered immune responses [6]. As such, clinicians may be disoriented by the number of surgical procedures and conditions that may require an AP. As a general principle, the prescription of prophylactic antibiotics should be reasonable and weighted on the basis of a risk–benefit analysis, with consideration also given to the consequences of unnecessary administrations on increases in AMR among the general population [7]. The last annual national report of the Italian drug agency [8] painted a worrying picture of AMR, noting how the high consumption of antibiotics is increasing the spread of AMR, and estimating that antibiotics are over-prescribed more than 25% of the time. Another study on elective minor surgical procedures found that in approximately half of all cases, the antibiotic prophylaxis was not consistent with the guidelines [9]. No specific data have been reported, however, about the use of antibiotics in dentistry and, more importantly, on the specific indication for prescriptions, distinguishing between AP for a cardiac condition, AP to prevent SSI, and therapeutic prescription [10]. For this reason, it is important to investigate the prescribing habits of dentists and to assess whether antibiotic prescriptions are inappropriate with respect to current evidences. By comparing results from other national surveys, these data may provide the basis for elaborating international guidelines and antibiotic stewardship programs on the correct use of AP to prevent SSIs in dentistry.

The objective of this study was to collect information on the prescribing habits of a sample of Italian dentists with respect to antibiotic prophylaxis for the most common oral surgical procedures.

## 2. Results

At the survey’s conclusion, 169 replies were collected with a response rate of 6.76%. The modality of the questionnaire administration did not allow the characteristics of non-respondents to be disclosed. Demographic data of respondents are reported in Table 1. The age of respondents ranged from 24 to 69 years, with a median age of 40 years. Most respondents were male (71%), with a Degree in Dentistry (82%) and professional experience of at least 5 years (80.5%). The most frequent specialization was Oral Surgery, though 69.2% of respondents had no specialization. Most replies came from Central Italy and from private practitioners (60.9). These data indicate that the respondents, in terms of their characteristics, can be considered representative of the general population.

**Figure 1:**
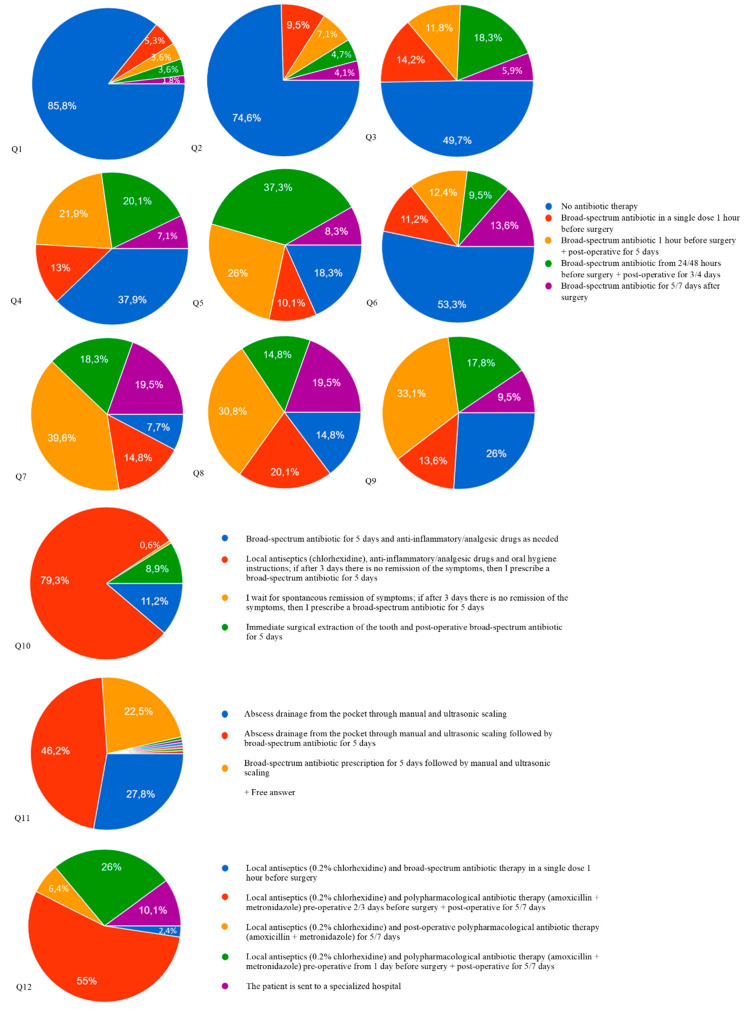
Graphs summarizing responses to specific questions about the use of prophylactic antibiotics to prevent SSI after the most common oral surgical procedures. Percentages are rounded to the first decimal place. The questions are reported in Figure 2.

A graphical representation of the answers to both the clinical cases and the short-form considerations are reported in Figure 1 and Table 2, respectively. Overall, only in a few cases was a strong agreement found among participants, with the responses varying considerably for most questions.

A moderate agreement on not prescribing antibiotics was found in Q1 (85.9%) and Q2 (74.6%), which concerned the extraction of a deciduous molar and a simple premolar extraction, respectively, in a 56-year-old patient with arterial hypertension.

The percentage of “no antibiotic” response decreased in Q3 (49.7%), Q4 (37.9%), Q5 (18.3%) and Q6 (53.3%), which concerned cases of non-surgical single or multiple extractions in patients with well-controlled systemic diseases, and in Q9 (26%), where the patient had a perioperative uncontrolled blood glucose (BG). Moreover, an agreement was not found on timing and posology.

In Q7 (the surgical extraction of an impacted wisdom tooth), only 7.7% of participants would not prescribe antibiotics or would prescribe a single preoperative dose (14.8%), whereas 77.4% of respondents would prescribe pre- and post-operative antibiotics or exclusively postoperative antibiotics.

As for Q8 (implant surgery), responses were also heterogeneous, with only 20.1% of clinicians prescribing exclusively one preoperative dose, and 14.8% not prescribing any antibiotic. The remaining respondents opted for postoperative AP with or without preoperative doses.

In Q10 (the pericoronitis case), 79.3% of the participants chose to use local antiseptics and anti-inflammatories and to re-evaluate after 3 days, avoiding antibiotics in the first instance. On the contrary, in Q11, the majority of respondents (46.2%) agreed in prescribing a 5-day antibiotic therapy following drainage and manual/ultrasonic debridement of the pocket; 22.5% would prescribe antibiotics before scaling, whereas only 27.8% would perform drainage and scaling without antibiotics.

In Q12, most respondents opted for perioperative AP starting from 2 to 3 days (55%) or 1 day (26%) before the extraction; 6.4% would prescribe only postoperative AP, 2.4% would prescribe only a single preoperative dose, and 10.1% would send the patient to a specialized hospital.

In the third section, short-form considerations were proposed on specific issues. C1 and C2 were meant to assess the relationship between oral hygiene conditions and antibiotic prescriptions. In C1, clinicians were asked whether they performed an oral hygiene session (OHS) before non-surgical extraction: 57.4% performed an OHS only in cases of abundant plaque and calculus deposits, 27.8% always performed an OHS, 14.2% did not always perform an OHS even in cases of scarce oral hygiene, and 0.6% never performed an OHS before non-surgical extractions. In C2, 57.4% of respondents considered poor oral hygiene and low patient compliance an indication for AP. On the contrary, 82.2% of respondents did not consider the use of haemostatic materials in post-extractive sockets to be an indication for the prescription of antibiotics (C3), whereas 83.4% prescribed postoperative AP in the case of a ridge preservation procedure with bone substitutes (C4). In C5, 42.6% of respondents considered postoperative antibiotics useful after the non-surgical extraction of teeth with periapical granuloma. More than half of respondents (54.4%) referred to having prescribed AP despite considering it unnecessary (C6-C7). The principal reasons for unnecessary prescriptions included concerns regarding disputes or complaints from the patients (44.7%), post-operative pain management (39.4%), patient’s request (34%), impossibility to visit the patient (26.6%), and apprehensive patients (18.1%). Interestingly, in the free answers, some reported insistence by other clinicians.

Finally, 74.6% of respondents referred to having attended courses or having read scientific papers on the use of antibiotics in dentistry during the last two years (C8). However, only 26.6% of respondents considered themselves fully informed on the issue of AMR and prescription appropriateness, with 66.9% considering themselves informed enough, but in need of more information (C9). Significantly, most respondents considered inappropriate antibiotic prescriptions as medium (26%), very (47.3%) or extremely (21.3%) widespread (C10).

## 3. Discussion

Antibiotics represent an inestimable resource, helping to save the lives of millions worldwide from potentially fatal infectious diseases. Several guidelines and programs have been developed to prevent AMR phenomena by reducing the inappropriate use of antibiotics. Notwithstanding, inappropriate prescriptions are still widespread [11]. In this context, the results of this study indicate that prophylactic administrations of antibiotics in dentistry are mainly based on subjective considerations, and only occasionally does an agreement exist on indications, dosage and timing.

A general agreement was found in not prescribing AP after non-surgical extractions in healthy patients, whereas different approaches were followed for patients with comorbidities. There are few studies on the efficacy of AP for the extraction of normally erupted teeth. In a recent retrospective study [12] on 418 patients who underwent non-surgical extractions, 280 received antibiotics, whereas 138 did not receive any medication. Only 12 total cases of alveolitis were reported (2.87%), with half of those cases receiving antibiotics. It was concluded that antibiotics do not prevent post-extractive alveolitis after the extraction of erupted teeth. These observations are supported by an RCT [13], which confirmed that antibiotics are not effective in the prevention of SSI after non-surgical extractions. It is important to note that, with the exception of cardiac conditions at the highest risk of infective endocarditis [14,15], common cardiovascular conditions such as arterial hypertension are not an indication for AP [16]. Moreover, there are currently no evident correlations between old age and an increased risk of post-extractive infections [12].

In the case of an extraction of a lower third molar with complete bony impaction, the vast majority of respondents (77.4%) would prescribe pre- and post-operative or only postoperative antibiotics, whereas only 7.7% of participants would not prescribe antibiotics or would prescribe a single preoperative dose (14.8%). The use of antibiotics after surgical extractions is still controversial. In a randomized, double-blind, placebo-controlled trial including 118 patients undergoing the surgical extraction of impacted third molars, Arteagoitia et al. [17] found no significant differences in SSI incidence between the treated group (2 g amoxicillin/125 mg clavulanic acid 2 h before the surgery and postoperatively twice a day for 4 days) and the placebo group. These data are supported by the observation that the risk of infection related to the extraction of impacted lower third molars is, in general, lower than 6% [18], leading some authors to discourage the routine use of antibiotics in healthy patients in the absence of pre-existing infections or complicated and long-lasting procedures. On the contrary, a recent Cochrane systematic review [19] reported a lower incidence of postsurgical infectious complications after third molar extractions in patients treated with antibiotics, compared to a placebo. In 21 of the 23 reviewed studies, only impacted third molars were included. According to the authors, antibiotics may reduce the risk of infection by approximately 66%, although this is with a low-certainty evidence and no definitive conclusion about the best timing of administration. Nevertheless, as already noted by Sancho-Puchades [20], it is often difficult to distinguish between infectious and non-infectious complications, because pain, acute swelling and trismus may be secondary both to the surgical trauma or to infections. It should, therefore, be considered a possible diagnostic bias among different studies due to the presence of common postoperative complications misdiagnosed as postoperative infections.

As for implant surgery, different studies have indicated that a single preoperative dose of a wide-spectrum antibiotic can reduce early implant loss (1.8% vs. 5.6%) [21] but does not prevent SSI [21,22,23]. Only 20.1% of the participants opted for a single preoperative dose; similar findings have been reported in a previous survey [24]. Cross-sectional studies conducted in other countries have shown that an exclusively preoperative prophylaxis was prescribed by 32.4% of a Dutch sample [25] and 5.73% of a Spanish sample [26]. Based on the current evidence, postoperative antibiotics may not be shown to prevent early implant failures and SSIs in healthy patients undergoing implant surgery [26]. Furthermore, studies and reviews suggest that in the case of “straightforward” implant surgery in healthy patients, antibiotics should not be prescribed [21,27,28]. However, despite this, a review of epidemiological investigations showed that three quarters of participants routinely prescribe antibiotics in implant surgery procedures in healthy patients [29].

Diabetes mellitus is commonly considered an independent risk factor for SSIs following surgical procedures [30]. Despite the paucity of good evidence in the dental field, most clinicians assume a higher risk for postoperative infections in diabetic patients. This putative excess risk is followed by recommendations to prescribe antibiotic prophylaxis for diabetics undergoing dental osseous surgery [31]. There is less evidence concerning the necessity of AP to prevent SSI following tooth extractions in diabetic patients. In a prospective observational study, Krishnan et al. [32] found a slight, but not significant increase in the risk of postoperative infections in type 2 diabetic patients undergoing tooth extractions with no AP, compared to healthy patients; other factors including glycemia, glycated haemoglobin, age and number of extractions were not associated with an increased risk of infection. Similar results were observed for insulin-dependent diabetic patients following tooth extraction without AP, with a small, though not statistically significant, increase in wound infections compared to healthy patients [33]. Based on the current evidence, the routine use of antibiotics in diabetic patients after single or multiple extractions does not seem to be justified, though a closer postoperative observation is required for these patients. Nevertheless, in the case presented, only 26% of respondents would not prescribe an AP.

Pericoronitis and periodontal abscess are common conditions that rarely present with systemic signs and symptoms. In the pericoronitis case (Q10), 79.3% of the participants chose local antiseptics and anti-inflammatories with re-evaluation after 3 days. In Q11, most respondents (46.2%) agreed on performing drainage and debridement of the pocket associated with postoperative antibiotics for 5 days, whereas 27.8% would only perform drainage and instrumentation, and 22.5% would perform antibiotic therapy before drainage and instrumentation. Among the free answers, some indicated the use of topical antibiotics after the socket debridement. The Clinical Practice Guideline by the European Federation of Periodontology [34] does not recommend the routine use of systemic antibiotics as an adjunct to subgingival debridement in patients with periodontitis, though periodontal abscesses are not specifically discussed. It was reported that the use of an antibiotic may be indicated when drainage/debridement is not possible and if the infection can spread and there are signs and symptoms of systemic involvement such as fever, lymphadenopathy, trismus etc. [35,36]. According to literature indications, the use of antibiotics in the case presented seems unjustified, since both the cause (subgingival plaque and calculus) and the effect (abscess) can be treated by drainage and instrumentation. However, antibiotics can be used as an adjuvant therapy in addition to the mechanical therapy in the case of severe infections, or as an attack therapy followed by the mechanical therapy when symptoms decrease after 2–3 days.

Q12 proposes the case of a non-surgical extraction in a non-oncological patient (n-OP) who regularly takes oral bisphosphonates (BP). In accordance with current guidelines, most respondents agreed on the need for adequate AP against aerobic and anaerobic pathogens [37,38]. Osteonecrosis of the jaws (ONJ) following tooth extraction is relatively rare among patients that take oral BP. On the contrary, dental extractions can be a precipitating factor for ONJ in 52–61% of patients taking intravenous BP. The case of patients under BP therapy is an exemplary situation in which, despite the low risk of the complication, AP is recommended due to the severity of the complication itself. To achieve a greater broad-spectrum action, a combination of amoxicillin 1 g t.i.d. and metronidazole 500 mg t.i.d. has been recommended, although no definitive data are available indicating the optimal timing, preoperative, postoperative or both.

It is interesting that most respondents considered scarce oral hygiene as an indication for postoperative AP (C1), and that an oral hygiene session is not always performed before surgical procedures (C2). This means that many prescriptions could be avoided by performing routine oral hygiene procedures and instructing the patients before surgical interventions. Though it is good practice and common sense to perform an oral hygiene session in the case of plaque and calculus deposits, no study has investigated the relation between oral hygiene and postoperative infections in dentistry, and there is no evidence that antibiotics can reduce the risk of infection in cases of low compliance and scarce oral hygiene. Similarly, since the use of hemostatic materials or sutures (C3) in non-surgical extractions does not increase the risk of SSI [12], the use of antibiotics is not justified, as indicated by 82.2% of the participants. Conversely, in the case of ridge preservation procedures with bone grafting (C4), 83.4% of participants agreed on the need for antibiotics, though literature data are scarce and conflicting on this issue [39,40], and it has been suggested that a single preoperative dose (2 or 3 g of amoxicillin) would be sufficient to prevent SSI after bone regeneration procedures [41].

The use of antibiotics after the extraction of teeth with periapical lesions is controversial. A systematic review has observed a high survival rate of implants placed in post-extraction sites of teeth with periapical/periodontal lesions after an adequate chemical–mechanical disinfection of the alveolus, whereas the effectiveness of local or systemic antibiotics is not proven [42].

Notably, 54.4% of the participants reported to have prescribed antibiotics they did not consider necessary (C7). Most respondents prescribed unnecessary antibiotics as a defensive medicine (options a, c, f). Interestingly, 11 of the 94 respondents indicated an insistence from colleagues (tutor, employer) or other specialists (cardiologist, general practitioner).

Finally, the participants reported that in the last 2 years they had attended courses on the correct use of antibiotics in dentistry (74.6% in C8), and that they were sufficiently informed about AMR (66.9% in C9). Significantly, according to the participants, the prescriptive inappropriateness has medium to extreme diffusion (94.6% in C10).

The results of this study, on the whole, confirm that the choice to prescribe an antibiotic is often based on subjective judgements and the biases of dentists. Several dentists prescribe unnecessary antibiotics as a defensive medicine or due to an overestimation of the risk and severity of postoperative complications. This can only be partially justified by the lack of guidelines, since some evidence already exists supporting a more reasonable use of antibiotics. These data are useful for identifying the cases in which the need for AP is still controversial, and could provide the starting point for clinical studies investigating the real usefulness of antibiotics for specific dental procedures.

This study has some limitations. Firstly, there was a relatively limited number of participants. Although the characteristics of the respondents indicate an adequate representativeness of the sample with respect to the general population of dental practitioners, the small number may limit the ability to generalize of the results. Furthermore, the study design and spread modality did not allow the characteristics of non-respondents to be identified. Finally, the limited number of answer options to the cases presented could have excluded other possible approaches of clinicians.

## 4. Materials and Methods

**Figure 2:**
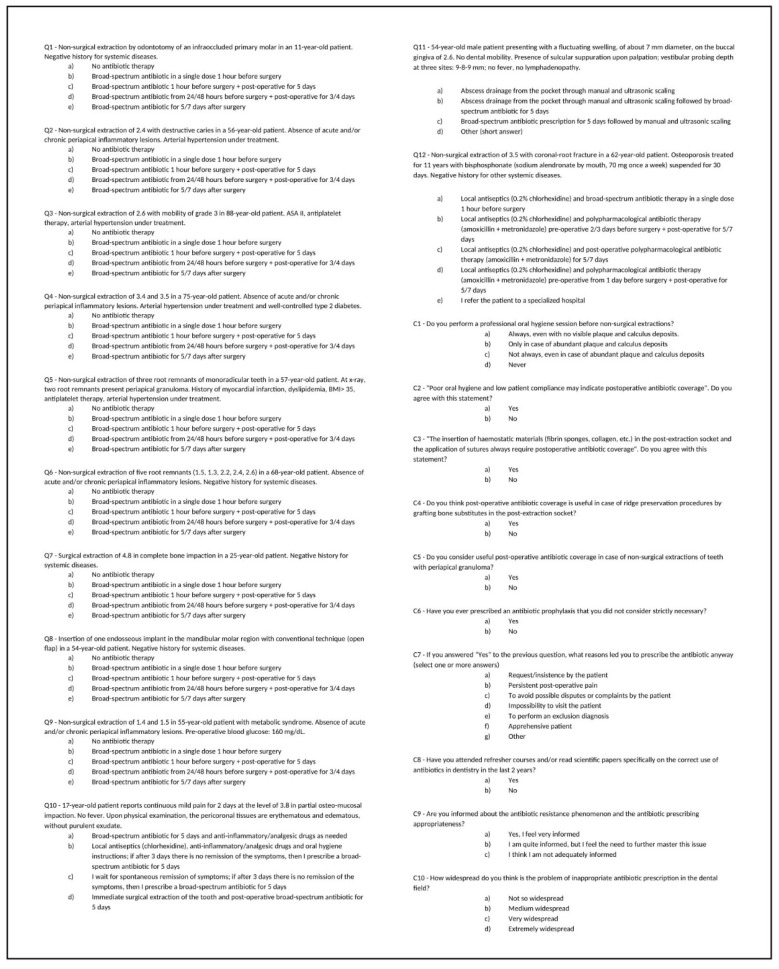
Questionnaire including clinical cases (Q1–Q12) and short-form considerations (C1–C10) about the use of antibiotics in specific dental procedures.

A survey was conducted among a non-random sample of Italian dentists through the administration of an anonymous questionnaire. The questionnaire (see Supplementary Materials) was prepared on the Google Forms platform and shared on different Italian dental social media and dental associations from June to December 2020. Considering the success and spread of new social media among dental clinicians, it was decided to share the questionnaire through these platforms in order to reach the highest number of participants from all regions, including both general practitioners and specialists. Recording the number of members of social media groups and dental associations at the start of the survey, a total of 2500 recipients was calculated. The opening page of the questionnaire was meant to provide information about the survey’s intentions and the anonymous nature of the study. At that point, the recipients could leave or give their consent to continue with the questionnaire. The survey was structured in three parts. In the first section, demographic data were collected, including age, gender, education, professional experience and region. In the second section (Figure 2), clinical scenarios were presented, including the most common surgical procedures performed by dentists, and participants were asked to choose the most suitable AP or therapy regimen among those proposed. Cases of single or multiple non-surgical extractions were proposed in Q1, 2, 3, 4, 5, 6, 9 and 12. Q7 and Q8 reported the surgical extraction of an impacted wisdom tooth and the case of a single implant placement, respectively. Q10 and Q11 reported cases of pericoronitis and periodontal abscess, respectively, both without systemic signs and symptoms, and Q12 proposed the case of a non-surgical extraction in a non-oncological patient (n-OP) who regularly takes oral bisphosphonates (BP). Clinical variables included the patients’ age, systemic conditions, and the type/number of surgical procedures. The standard response options for most questions (except in Q10, 11 and 12) included four possible timings and posology of broad-spectrum antibiotics, or the possibility of “no antibiotics”. Short-form considerations (C1–10) about the use of antibiotics in specific procedures, and about AMR awareness, were also presented. For two questions (Q11 and C7) there was an opportunity for respondents to write a free answer.

The answers received have been assessed through the Google Forms percentage report. Descriptive statistics, including mean and percentage, were calculated for each variable.

## 5. Conclusions

The results of this survey indicate that the choice to prescribe prophylactic antibiotics to prevent postoperative complications after oral surgical procedures is mostly based on the subjective considerations of clinicians. Considering the increasing phenomena of antimicrobial resistance, it is essential that clinicians prescribe prophylactic antibiotics only when strictly necessary, since many prescriptions could be avoided based on the current evidence. On the other hand, scientific societies should elaborate specific guidelines for the correct use of prophylactic antibiotics to prevent SSIs, specifically addressing the most controversial issues. To this end, more randomized controlled trials investigating the need of antibiotics prophylaxis for the most common oral surgical procedures are urgently required.

## Figures and Tables

**Table 1:** Demographic data of participants.

		N°	%
Gender	Male	120	71
	Female	49	29
Educational qualification	Degree in Dentistry	140	82.84
	Degree in Medicine and Surgery specialized in Odontostomatology	20	11.83
	Degree in Medicine and Surgery	9	5.33
Years of professional experience	<5	33	19.53
	5–10	46	27.22
	10–15	13	7.69
	>15	77	45.56
Dental speciality	Oral surgery	43	25.44
	Orthodontics	8	4.73
	Pediatric Dentistry	4	2.37
	None	117	69.23
Region	Northern Italy	40	23.67
	Central Italy	95	56.21
	Southern and Insular Italy	34	20.12
Employment	Private practice	103	60.95
	Hospital clinic	12	7.1
	Both	54	31.95

**Table 2:** Responses to questions about the use of antibiotics in specific clinical scenarios and antibiotic resistance awareness. Percentages are rounded to the first decimal place. Questions are reported in Figure 2.

Question	Answer	%
C1	Always, even with no visible plaque and calculus deposits	27.8
	Only in case of abundant plaque and calculus deposits	57.4
	Not always, even in case of abundant plaque and calculus deposits	14.2
	Never	0.6
C2	Yes	57.4
	No	42.6
C3	Yes	17.8
	No	82.2
C4	Yes	83.4
	No	16.6
C5	Yes	42.6
	No	57.4
C6	Yes	54.4
	No	45.6
C8	Yes	74.6
	No	25.4
C9	Yes, I feel very informed	26.6
	I am quite informed but I feel the need to further master this issue	66.9
	I think I am not adequately informed	6.5
C10	Not so widespread	5.3
	Medium widespread	26
	Very widespread	47.3
	Extremely widespread	21.3

## Data Availability

The data presented in this study are available on request from the corresponding author.

## Supplementary Materials

The following Supplementary Materials are available online at https://www.mdpi.com/article/10.3390/antibiotics10080949/s1, PDF file: Questionnaire.
