# Supplementary material for: Antibiotics to Prevent Surgical Site Infection (SSI) in Oral Surgery: Survey among Italian Dentists
Source: Antibiotics (Basel). 2021 Aug 6;10(8):949. doi: 10.3390/antibiotics10080949 (PMC8388912; doi:10.3390/antibiotics10080949)
Supplement: Supplementary file 1 [file antibiotics-10-00949-s001.zip › antibiotics-1300388-supplementary.pdf]

ANTIBIOTIC PRESCRIPTION SURVEY

Q1 - Non-surgical extraction by odontotomy of an infraoccluded primary molar in an 11-year-old patient. Negative history for systemic diseases.

a) No antibiotic therapy
b) Broad-spectrum antibiotic in a single dose 1 hour before surgery
c) Broad-spectrum antibiotic 1 hour before surgery + post-operative for 5 days
d) Broad-spectrum antibiotic from 24/48 hours before surgery + post-operative for 3/4 days
e) Broad-spectrum antibiotic for 5/7 days after surgery

Q2 - Non-surgical extraction of 2.4 with destructive caries in a 56-year-old patient. Absence of acute and/or chronic periapical inflammatory lesions. Arterial hypertension under treatment.

a) No antibiotic therapy
b) Broad-spectrum antibiotic in a single dose 1 hour before surgery
c) Broad-spectrum antibiotic 1 hour before surgery + post-operative for 5 days
d) Broad-spectrum antibiotic from 24/48 hours before surgery + post-operative for 3/4 days
e) Broad-spectrum antibiotic for 5/7 days after surgery

Q3 - Non-surgical extraction of 2.6 with mobility of grade 3 in 88-year-old patient. ASA II, antiplatelet therapy, arterial hypertension under treatment.

a) No antibiotic therapy
b) Broad-spectrum antibiotic in a single dose 1 hour before surgery
c) Broad-spectrum antibiotic 1 hour before surgery + post-operative for 5 days
d) Broad-spectrum antibiotic from 24/48 hours before surgery + post-operative for 3/4 days
e) Broad-spectrum antibiotic for 5/7 days after surgery

Q4 - Non-surgical extraction of 3.4 and 3.5 in a 75-year-old patient. Absence of acute and/or chronic periapical inflammatory lesions. Arterial hypertension under treatment and well-controlled type 2 diabetes.

a) No antibiotic therapy
b) Broad-spectrum antibiotic in a single dose 1 hour before surgery
c) Broad-spectrum antibiotic 1 hour before surgery + post-operative for 5 days
d) Broad-spectrum antibiotic from 24/48 hours before surgery + post-operative for 3/4 days
e) Broad-spectrum antibiotic for 5/7 days after surgery

Q5 - Non-surgical extraction of three root remnants of monoradicular teeth in a 57-year-old patient. At x-ray, two root remnants present periapical granuloma. History of myocardial infarction, dyslipidemia, BMI> 35, antiplatelet therapy, arterial hypertension under treatment.

a) No antibiotic therapy
b) Broad-spectrum antibiotic in a single dose 1 hour before surgery
c) Broad-spectrum antibiotic 1 hour before surgery + post-operative for 5 days
d) Broad-spectrum antibiotic from 24/48 hours before surgery + post-operative for 3/4 days
e) Broad-spectrum antibiotic for 5/7 days after surgery

Q6 - Non-surgical extraction of five root remnants (1.5, 1.3, 2.2, 2.4, 2.6) in a 68-year-old patient. Absence of acute and/or chronic periapical inflammatory lesions. Negative history for systemic diseases.

a) No antibiotic therapy
b) Broad-spectrum antibiotic in a single dose 1 hour before surgery
c) Broad-spectrum antibiotic 1 hour before surgery + post-operative for 5 days
d) Broad-spectrum antibiotic from 24/48 hours before surgery + post-operative for 3/4 days
e) Broad-spectrum antibiotic for 5/7 days after surgery

Q7 - Surgical extraction of 4.8 in complete bone impaction in a 25-year-old patient. Negative history for systemic diseases.

a) No antibiotic therapy
b) Broad-spectrum antibiotic in a single dose 1 hour before surgery
c) Broad-spectrum antibiotic 1 hour before surgery + post-operative for 5 days
d) Broad-spectrum antibiotic from 24/48 hours before surgery + post-operative for 3/4 days
e) Broad-spectrum antibiotic for 5/7 days after surgery

Q8 - Insertion of one endosseous implant in the mandibular molar region with conventional technique (open flap) in a 54-year-old patient. Negative history for systemic diseases.

a) No antibiotic therapy
b) Broad-spectrum antibiotic in a single dose 1 hour before surgery
c) Broad-spectrum antibiotic 1 hour before surgery + post-operative for 5 days
d) Broad-spectrum antibiotic from 24/48 hours before surgery + post-operative for 3/4 days
e) Broad-spectrum antibiotic for 5/7 days after surgery

Q9 - Non-surgical extraction of 1.4 and 1.5 in 55-year-old patient with metabolic syndrome. Absence of acute and/or chronic periapical inflammatory lesions. Pre-operative blood glucose: 160 mg/dL.

a) No antibiotic therapy
b) Broad-spectrum antibiotic in a single dose 1 hour before surgery
c) Broad-spectrum antibiotic 1 hour before surgery + post-operative for 5 days
d) Broad-spectrum antibiotic from 24/48 hours before surgery + post-operative for 3/4 days
e) Broad-spectrum antibiotic for 5/7 days after surgery

Q10 - 17-year-old patient reports continuous mild pain for 2 days at the level of 3.8 in partial osteo-mucosal impaction. No fever. Upon physical examination, the pericoronal tissues are erythematous and edematous, without purulent exudate.

a) Broad-spectrum antibiotic for 5 days and anti-inflammatory/analgesic drugs as needed
b) Local antiseptics (chlorhexidine), anti-inflammatory/analgesic drugs and oral hygiene instructions; if after 3 days there is no remission of the symptoms, then I prescribe a broad-spectrum antibiotic for 5 days
c) I wait for spontaneous remission of symptoms; if after 3 days there is no remission of the symptoms, then I prescribe a broad-spectrum antibiotic for 5 days
d) Immediate surgical extraction of the tooth and post-operative broad-spectrum antibiotic for 5 days

Q11 - 54-year-old male patient presenting with a fluctuating swelling, of about 7 mm diameter, on the buccal gingiva of 2.6. No dental mobility. Presence of sulcular suppuration upon palpation; vestibular probing depth at three sites: 9-8-9 mm; no fever, no lymphadenopathy.

a) Abscess drainage from the pocket through manual and ultrasonic scaling
b) Abscess drainage from the pocket through manual and ultrasonic scaling followed by broad-spectrum antibiotic for 5 days
c) Broad-spectrum antibiotic prescription for 5 days followed by manual and ultrasonic scaling
d) Other (short answer)

Q12 - Non-surgical extraction of 3.5 with coronal-root fracture in a 62-year-old patient. Osteoporosis treated for 11 years with bisphosphonate (sodium alendronate by mouth, 70 mg once a week) suspended for 30 days. Negative history for other systemic diseases.

a) Local antiseptics (0.2% chlorhexidine) and broad-spectrum antibiotic therapy in a single dose 1 hour before surgery

b) Local antiseptics (0.2% chlorhexidine) and polypharmacological antibiotic therapy (amoxicillin + metronidazole) pre-operative 2/3 days before surgery + post-operative for 5/7 days
c) Local antiseptics (0.2% chlorhexidine) and post-operative polypharmacological antibiotic therapy (amoxicillin + metronidazole) for 5/7 days
d) Local antiseptics (0.2% chlorhexidine) and polypharmacological antibiotic therapy (amoxicillin + metronidazole) pre-operative from 1 day before surgery + post-operative for 5/7 days
e) I refer the patient to a specialized hospital

C1 - Do you perform a professional oral hygiene session before non-surgical extractions?

a) Always, even with no visible plaque and calculus deposits.
b) Only in case of abundant plaque and calculus deposits
c) Not always, even in case of abundant plaque and calculus deposits
d) Never

C2 - "Poor oral hygiene and low patient compliance may indicate postoperative antibiotic coverage". Do you agree with this statement?

a) Yes
b) No

C3 - "The insertion of haemostatic materials (fibrin sponges, collagen, etc.) in the post-extraction socket and the application of sutures always require postoperative antibiotic coverage". Do you agree with this statement?

a) Yes
b) No

C4 - Do you think post-operative antibiotic coverage is useful in case of ridge preservation procedures by grafting bone substitutes in the post-extraction socket?

a) Yes
b) No

C5 - Do you consider useful post-operative antibiotic coverage in case of non-surgical extractions of teeth with periapical granuloma?

a) Yes
b) No

C6 - Have you ever prescribed an antibiotic prophylaxis that you did not consider strictly necessary?

a) Yes
b) No

C7 - If you answered “Yes” to the previous question, what reasons led you to prescribe the antibiotic anyway (select one or more answers)

a) Request/insistence by the patient
b) Persistent post-operative pain
c) To avoid possible disputes or complaints by the patient
d) Impossibility to visit the patient
e) To perform an exclusion diagnosis
f) Apprehensive patient
g) Other

C8 - Have you attended refresher courses and/or read scientific papers specifically on the correct use of antibiotics in dentistry in the last 2 years?

a) Yes

b) No

C9 - Are you informed about the antibiotic resistance phenomenon and the antibiotic prescribing appropriateness?

a) Yes, I feel very informed
b) I am quite informed, but I feel the need to further master this issue
c) I think I am not adequately informed

C10 - How widespread do you think is the problem of inappropriate antibiotic prescription in the dental field?

a) Not so widespread
b) Medium widespread
c) Very widespread
d) Extremely widespread

****
